# Supplementary material for: Perceptions and Attitudes of Correctional Staff Toward ADHD—A Challenging Disorder in Everyday Prison Life
Source: Front Psychiatry. 2021 Jan 28;11:600005. doi: 10.3389/fpsyt.2020.600005 (PMC7901975; doi:10.3389/fpsyt.2020.600005)
Supplement: Supplementary file 1 [file Table_1.DOCX]

**Supplementary Table 1**

Survey to obtain additional biographical data and previous experiences with mental illness

Gender

Age

*Employment in the penal system*

1. How long have you been working in the prison system?

*Training*

1. What is the highest level of education or vocational training that you have completed?

2. Are you in possession of an individual certificate of proficiency (EFZ)?

3. Have you begun and/or completed training as a specialist for correctional services?

4. Have you begun and/or completed leadership training for correctional officers?

5. Have you begun and/or completed postgraduate training courses?

(…)

*Past experiences with mental disorders*

1. Did you learn about mental illness during your training? (Yes/No)

If so, do you find the knowledge you acquired sufficient for your everyday work? (Yes/ No)

2. Were you during your courses trained to deal with mentally ill offenders? (Yes/ No)

If so, do you think that this training was adequate and prepared you well for the situations you encounter in everyday life? (Yes/ No)

3. Do you know anybody in your personal environment who has consulted a psychiatrist or psychotherapist because of a mental illness? (Yes/No)

4. Do you know anybody in your personal environment who has been treated in a psychiatric clinic due to a mental illness? (Yes/No)

Did this affect:

A friend... (Yes/No)

A family member… (Yes/No)

Another relative…(Yes/No)

Yourself?...(Yes/No)

5. Have you yourself ever had the feeling of suffering from a mental disorder?... (Yes/No)
